# Epithelial-specific histone modification of the *miR-96/182* locus targeting *AMAP1* mRNA predisposes p53 to suppress cell invasion in epithelial cells

**DOI:** 10.1186/s12964-018-0302-6

**Published:** 2018-12-04

**Authors:** Haruka Handa, Ari Hashimoto, Shigeru Hashimoto, Hirokazu Sugino, Tsukasa Oikawa, Hisataka Sabe

**Affiliations:** Department of Molecular Biology, Graduate School of Medicine, Hokkaido University, North 15, West 7, Kita-ku, Sapporo, Hokkaido 060-8638 Japan

**Keywords:** AMAP1, miRNA, miR-96/182, p53, Epithelial cell invasion

## Abstract

**Background:**

*TP53* mutations in cancer cells often evoke cell invasiveness, whereas fibroblasts show invasiveness in the presence of intact *TP53*. AMAP1 (also called DDEF1 or ASAP1) is a downstream effector of ARF6 and is essential for the ARF6-driven cell-invasive phenotype. We found that AMAP1 levels are under the control of p53 (*TP53* gene product) in epithelial cells but not in fibroblasts, and here addressed that molecular basis of the epithelial-specific function of p53 in suppressing invasiveness via targeting AMAP1.

**Methods:**

Using MDA-MB-231 cells expressing wild-type and p53 mutants, we identified miRNAs in which their expression is controlled by normal-p53. Among them, we identified miRNAs that target *AMAP1* mRNA, and analyzed their expression levels and epigenetic statuses in epithelial cells and nonepithelial cells.

**Results:**

We found that normal-p53 suppresses *AMAP*1 mRNA in cancer cells and normal epithelial cells, and that more than 30 miRNAs are induced by normal-p53. Among them, miR-96 and miR-182 were found to target the 3′-untranslated region of *AMAP1* mRNA. Fibroblasts did not express these miRNAs at detectable levels. The ENCODE dataset demonstrated that the promoter region of the *miR-183-96-182* cistron is enriched with H3K27 acetylation in epithelial cells, whereas this locus is enriched with H3K27 trimethylation in fibroblasts and other non-epithelial cells. miRNAs, such as miR-423, which are under the control of p53 but not associated with *AMAP1* mRNA, demonstrated similar histone modifications at their gene loci in epithelial cells and fibroblasts, and were expressed in these cells.

**Conclusion:**

Histone modifications of certain miRNA loci, such as the *miR-183-96-182* cistron, are different between epithelial cells and non-epithelial cells. Such epithelial-specific miRNA regulation appears to provide the molecular basis for the epithelial-specific function of p53 in suppressing ARF6-driven invasiveness.

**Electronic supplementary material:**

The online version of this article (10.1186/s12964-018-0302-6) contains supplementary material, which is available to authorized users.

## Background

*TP53*, which encodes the tumor suppressor protein p53, is frequently mutated in human cancers. *TP53* mutations (i.e., loss of normal-p53 function) not only promote cell cycle progression, and cell growth and survival, but also evoke invasiveness and mesenchymal phenotypes in various cancer cells [1]. As for the inhibition of invasiveness by p53, the currently prevailing model indicates that p53 induces specific microRNAs (miRNAs) that target mRNAs of transcriptional factors that drive epithelial-mesenchymal transition (EMT-TFs), such as *ZEB1*, *SNAI1*, *SLUG* (*SNAI2*), and *BMI1* [2–4]. However, other types of cells, such as bona fide fibroblasts, demonstrate high invasiveness in the presence of intact *TP53,* and express these EMT-TFs [5]. Thus, some p53-miRNA axes might be specific to epithelial cells, although the molecular bases for such an epithelial-specific function of p53 remains largely elusive [6, 7].

AMAP1 (also called DDEF1 or ASAP1) is a downstream effector of the small GTP-binding protein ARF6 [8]. AMAP1 has multiple protein-protein interaction modules, and can interact with PRKD2 to promote integrin recycling [9], with EPB41L5 to disrupt E-cadherin-mediated cell-cell adhesion [10, 11], and also with cortactin and paxillin to remodel the actin-based cytoskeletal architecture [12]. Thus, AMAP1 is at the core for controlling cell invasiveness under the activity of ARF6, particularly during epithelial-mesenchymal transition (EMT). AMAP1, as well as ARF6, are expressed almost ubiquitously in various types of cells, although their enhanced expression is required to substantially drive cell invasive activity [13–15].

The *AMAP1* mRNA contains a 5′-terminal oligopyrimidine (TOP)-like sequence at its 5′-untranslated region (UTR), and hence is under the control of mTORC1 (S. Hashimoto et al., submitted). We here show that *AMAP1* mRNA is also under the control of p53, in which p53 appears to utilize miRNAs to target the 3’-UTR of this mRNA. Our analysis on the expression of p53-regulatable miRNAs provides insight into the molecular basis by which a specific p53-miRNA axis functions in epithelial cells but not in fibroblasts.

## Methods

### Cell lines

HEK293T cells, MDA-MB-231 cells, MCF7 cells, and BJ cells were purchased from American Type Culture Collection. MDA-MB-231 cells were cultured in 7.5% CO_2_ at 37 °C in a 1:1 mixture of Dulbecco’s modified Eagle medium (DMEM) (Invitrogen) and RPMI 1640 (Invitrogen), with 10% fetal calf serum (FCS) (HyClone) and 5% NU serum (BD Biosciences). The p53 derivatives of MDA-MB-231 cells were generated previously [16]. HEK293T cells, MCF7 cells and BJ cells were cultured at 37 °C in DMEM with 10% FCS (GE Healthcare, Illinois, USA). HMLE cells were gifted from Dr. Weinberg (Whitehead Institute, MIT, Cambridge, Massachusetts, USA) and cultured in Mammary Epithelial Cell Growth Medium (MEGM) (Lonza, Maryland, USA). HMLE cells expressing shp53 vectors were generated previously [11].

### MiRNA expression profiling

Cells were serum-starved for 16 h, and then left untreated or treated with TGFβ1 (2 ng/mL) for 2 h in the absence of FCS. Total cellular RNAs were then isolated using the QIAGEN RNeasy Mini Kit (QIAGEN, Netherland), according to the manufacturer’s instructions. Microarray analysis of miRNA expression was performed by Toray (Tokyo, Japan) using total cellular RNAs. Color visualization of the data was performed using Java TreeView software.

### RNA extraction and quantitative real-time polymerase chain reaction (RT-qPCR)

Total miRNA and mRNA were extracted from cultured cells using the miRNeasy mini kit (QIAGEN). TaqMan gene expression assays (Applied Biosystems:AB, Massachusetts, USA) were used for the analysis of AMAP1 mRNA, and TaqMan miRNA assays (AB) were for the analysis of hsa-miR-96 and hsa-miR182. Glyceraldehyde-3-phosphate dehydrogenase and the U6 small nuclear RNA were used as internal controls. These data were collected by 7300 Real Time PCR System (AB) and the ΔΔCt method was used for relative quantification [17].

### Dual luciferase reporter assay

HEK293T cells were transiently transfected using Lipofetamine LTX with pEZX-MT01 target reporter plasmids containing the full length of wild-type AMAP1 3′-UTR and its mutants lacking the miRNA target sequences, all of which were purchased from GeneCopeia (Maryland, USA). These cells were simultaneously transfected with oligonucleotide precursors of hsa-miR-96, hsa-miR-182, or hsa-miR-301a. A total of 5.0 × 10^4^ cells were plated onto a 24-well plate. Luciferase assays were performed 24 h after transfection using Dual-Luciferase Reporter Assay System (Promega, Madison, USA). Firefly luciferase activities were normalized to Renilla luciferase activity for each sample.

### Immunoblotting

P53 and β-actin were detected by antibodies purchased from commercial sources (mouse monoclonal anti-TP53, clone #2524, Cell Signaling; mouse monoclonal anti-β-actin, EMD Millipore, Massachusetts, USA). Rabbit polyclonal antibodies against AMAP1 were established as described previously [18]. Peroxidase-conjugated donkey antibodies against mouse or rabbit IgGs were purchased from Jackson ImmunoResearch Laboratories, Inc. All immunoblotting analyses were performed as described previously [16] using ECL Western detection reagents (GE Healthcare, Illinois, USA).

### Statistical analysis

All data were statistically analyzed by the unpaired *t*-test or Spearman rank correlation test using Prism 6.0 software (GraphPad Software, California, USA). A *P*-value of less than 0.05 was considered to indicate a statistically significant difference between two groups.

## Results

### *TP53* mutations enhance AMAP1 expression in epithelial cells

**Fig. 1:**
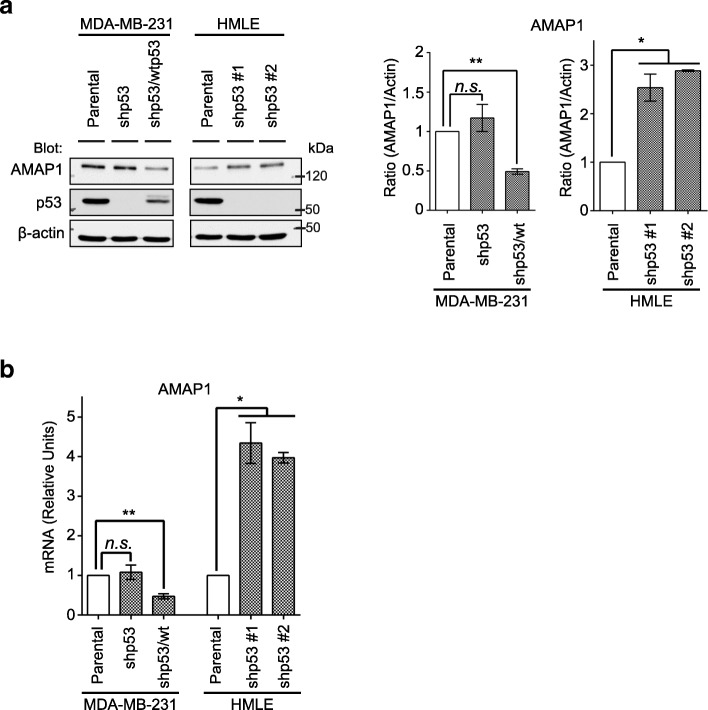
Loss of normal-p53 induces AMAP1 expression in MDA-MB-231 and HMLE cells. **a** Expression levels of AMAP1 and p53 were assessed by immunoblotting (left). The ratio of AMAP1/Actin was analyzed by densitometry and the results (right) are shown as the mean ± SEM (*n* = 2). *n.s.* means no significance. *P < 0.05. **b** Relative units of *AMAP1* mRNA were measured by quantitative-PCR. The mRNA level of MDA-MB-231 cells (parental) was used as a reference value for the other cell lines. The results are shown as the mean ± SEM (*n* = 3, HMLE series, *n* = 2, MDA-MB-231). *P < 0.05

MDA-MB-231 breast cancer cells express R280K mutant-p53 and have lost the other *TP53* allele [19]. We previously generated MDA-MB-231 cells in which endogenous mutant-p53 was silenced (shp53 cells), and shp53 cells expressing normal-p53 (shp53/wt cells) [16]. Western blot analysis of these cells demonstrated that AMAP1 protein levels were largely reduced in the presence of intact-p53 (Fig. 1a). AMAP1 mRNA levels were also significantly suppressed in the presence of intact-p53 (Fig. 1b).

To investigate whether the involvement of p53 in the suppression of AMAP1 expression is a general event, we next analyzed non-transformed cells. HMLE cells were generated by the immortalization of primary human normal mammary epithelial cell [20]. These HMLEs expressed the AMAP1 protein at a basal level, similar to that seen in shp53/wt cells (Fig. 1a); and shRNA-mediated silencing of p53 in these cells significantly enhanced AMAP1 expression, both at the protein and mRNA levels (Fig. 1a and b). Therefore, collectively, normal-p53 appeared to function to suppress AMAP1 levels in mammary epithelial cells, regardless of whether they are transformed or not.

### P53 induces miR-96 and miR-182 to target *AMAP1* mRNA

**Fig. 2:**
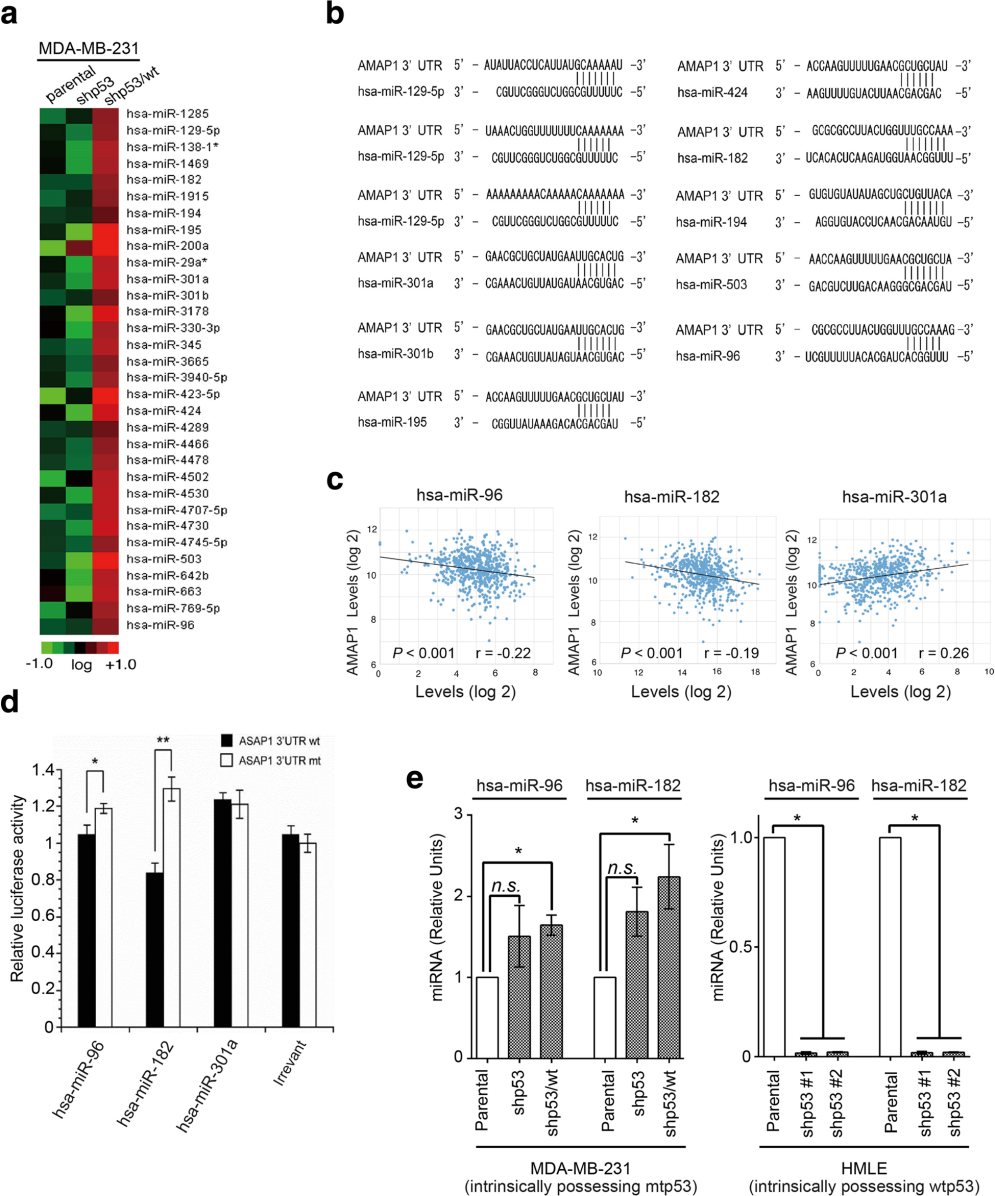
miR-96 and miR-182 target *AMAP1* mRNA. **a** Microarray analysis of gene expression in MDA-MB-231 cells (parental) and their p53 derivatives. Green, black, and red indicate transcript levels below, equal to, or above the mean, respectively, as shown at the bottom, in which gene expression intensities are represented in the log 2 scale. **b** Thirty-two miRNAs were expressed at higher levels in shp53/wt cells than in parental and shp53 cells (**a**), and 9 of them had homology to the 3’-UTR of the *AMAP1* mRNA. **c** Correlation diagrams between expression levels of miRNA candidates and *AMAP1* mRNA are shown. **d** The interactions among the *AMAP1* mRNA 3’-UTR (wt), or its mutant (mt) and *miR-96*, *miR-182*, or *miR-301a*, were assessed by luciferase reporter assay in HEK293T cells. Luciferase activities were measured and normalized to Renilla luciferase activity. The results are shown as the mean ± SEM (*n* = 3). *P < 0.05, **P < 0.01. **e** mRNA expression levels of two miRNA candidates in MDA-MB-231 cells and their p53 derivatives, and HMLE cells and their p53-knockdown cells were measured by quantitative RT-PCR. The results are shown as the mean ± SEM (*n* = 2). *n.s.* means no significance. *P < 0.05

The expression of various miRNAs is under the control of p53 [21]. To understand the possible mechanisms by which normal-p53 suppresses AMAP1 expression, we then analyzed the expression of miRNAs in MDA-MB-231 cells. In these experiments, we prepared miRNAs from cells cultured at sparse densities, to avoid density-dependent artefacts of miRNA preparation [22, 23]. Thirty-two different miRNAs were found to be expressed at significantly higher levels in shp53/wt cells than in the parental cells and shp53 cells (Fig. 2a). Nine of these miRNAs had nucleotide sequences that were complementary to the 3’-UTR of *AMAP1* mRNA (Fig. 2b). Among them, miR-96 and miR-182 levels were negatively correlated with the level of *AMAP1* mRNA in the TCGA RNASeq dataset on human primary breast tumors (Fig. 2c and Additional file 1: Figure S1). miR-96 and miR-182 are transcribed as the miR-183-96-182 cistron [24], and it was shown previously that p53 is responsible for the expression of this cistron by its direct binding [25].

We then generated a reporter gene, in which the firefly luciferase gene is fused to the *AMAP1* 3’-UTR, and found that miR-96 and miR-182 have the ability to target this 3’-UTR in a reconstituted system using HEK293T cells (Fig. 2d). As a control, we analyzed miR-301a. miR-301a was induced by normal-p53 and had a sequence complementary to the *AMAP1* 3’-UTR, whereas the TCGA database did not support a reciprocal association between this miRNA and *AMAP1* mRNA (Fig. 2a-c). We found that miR-301a is ineffective in targeting the *AMAP1* 3’-UTR in the reconstitution system (Fig. 2d). The suppression of miR-96 and miR-182 expression upon the loss of intact-p53 was also observed in HMLE cells (Fig. 2e), and we also confirmed statistically significant changes in *miR-96* and *miR-182* expression levels in MDA-MB-231 cells, depending on the p53 status (Fig. 2e). Therefore, miR-96 and miR-182 are likely to be involved in the suppression of *AMAP1* mRNA levels in response to normal-p53.

### The p53-*miR-96*/*182* axis is specific to epithelial cells and does not exist in fibroblasts

**Fig. 3:**
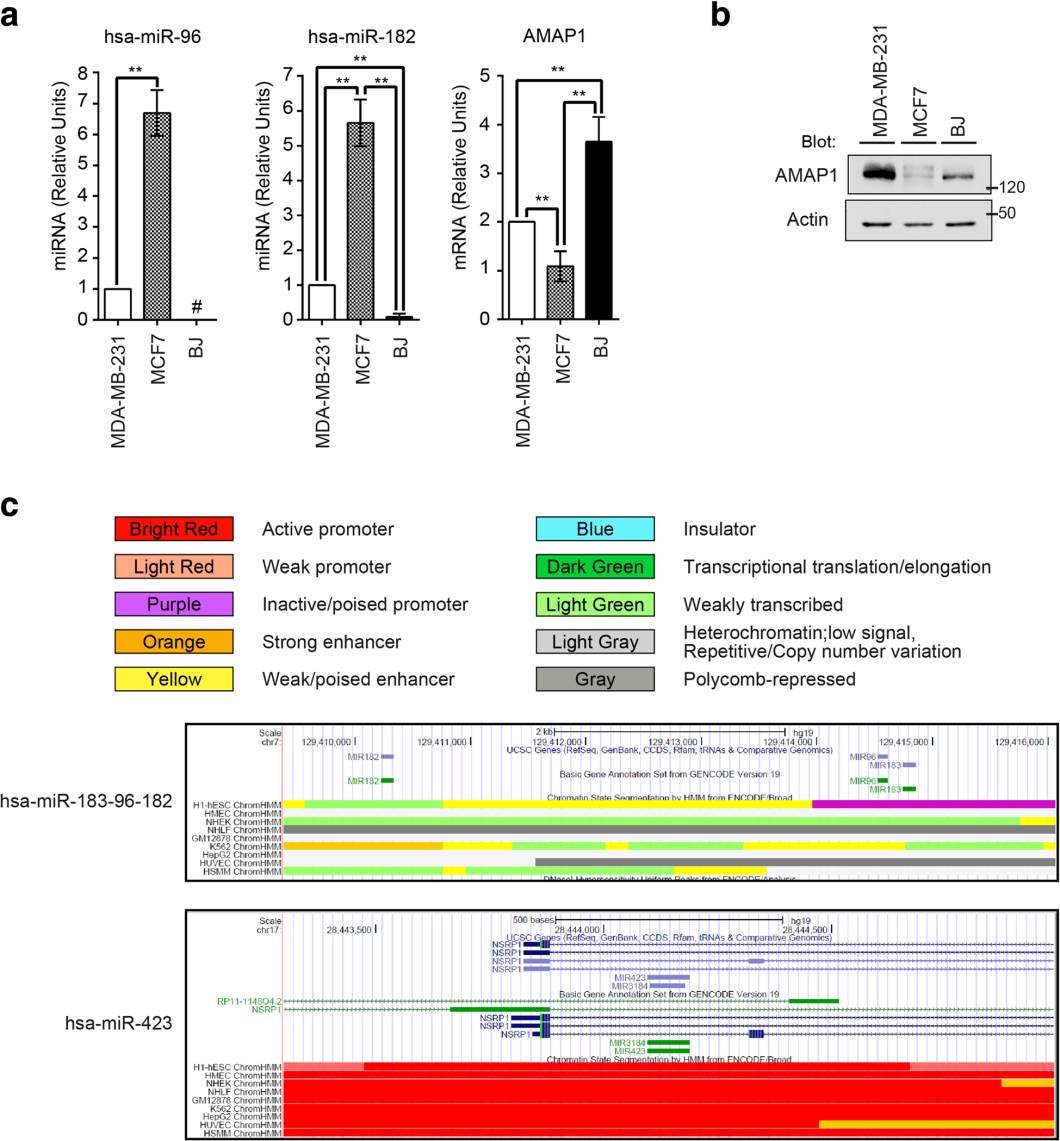
Expression and Epigenomic status of *miR-96*, *miR-182*, and *AMAP1.* **a** Expression levels of *miR-96*, *miR-182*, and *AMAP1* in MDA-MB-231 cells, MCF7 cells, and BJ cells were measured by quantitative RT-PCR. The unpaired *t*-test was performed (miR-96: *n* = 2; miR-182, AMAP1: *n* = 3) and the results are shown as the mean ± SEM. **P < 0.01. #: The mRNA level of BJ cell was under the detection limit. **b** Expression levels of AMAP1 were assessed by immunoblotting. **c** The ENCODE data of *miR-183-96-182* and *miR-423* loci by UCSC Genome Browser are shown. Definitions of the colors are given at the top. hESC: human embryonic stem cells, HMEC: human mammary epithelial cells, NHEK: normal human epidermal keratinocytes, NHLF: normal human lung fibroblasts, GM12878: B-lymphocyte transformed by Epstein-Barr virus, K562: highly undifferentiated and granurocytic myelogenous leukemia cells, HepG2:hephepatocytellular carcinoma cells, HUVEC: human umbilical vein endothelial cells, HSMM: human skeletal muscle myoblasts

MCF7 breast cancer cells express normal-p53 and are weakly invasive [26]. Consistently, MCF7 cells expressed the AMAP1 protein, as well as AMAP1 mRNA at much lower levels than MDA-MB-231 cells (Fig. 3a and b). On the other hand, normal human fibroblasts bearing intact *TP53*, such as BJ cells, expressed AMAP1 mRNA and protein at higher levels than MCF7 cells (Fig. 3a and b). We found that BJ cells do not notably express miR-96 and miR-182 (Fig. 3a).

A dataset of the ENCODE project [27, 28] then indicated that histone modification around the promoter region of the *miR-183-96-182* cistron is enriched with H3K27 trimethylation (H3K27me3) in fibroblasts, as well as in other non-epithelial cells, whereas this locus is enriched with H3K27 acetylation (H3K27ac) in epithelial cells (Fig. 3c). Thus, collectively, transcription of this cistron appeared to be epigenetically suppressed in fibroblasts, although it can be transcribed in epithelial cells.

### Not all p53-miRNA axes are specific to epithelial cells

The above results suggested an epithelial specificity of the p53-miR-96/182 axis. Normal p53 is known to induce many different types of miRNAs, as we have also shown above (see Fig. 2a). We were finally interested in understanding whether the induction of miRNAs by p53 is specific to epithelial cells, and does not occur in fibroblasts. *miR-423* is highly induced in the presence of p53 in MDA-MB-231 cells, but does not have nucleotide sequences complementary to the *AMAP1* mRNA 3’-UTR (see Fig. 2a and b). We found that histone modification of this locus was similar both in epithelial cells and fibroblasts, and was categorized as an active promoter (Fig. 3c). Likewise, histone modifications of the gene loci of some other miRNAs, which are under the control of normal-p53 in MDA-MB-231 cells, were not necessarily suppressed in fibroblasts (Additional file 2: Figure S2). Therefore, we concluded that not all p53-miRNA axes are specific only to epithelial cells.

## Conclusions

In this study, we showed that p53 acts to restrict *AMAP1* mRNA levels in epithelial cells, but not in fibroblasts. *AMAP1* mRNA contains a 5’-TOP-like sequence, and is hence under the control of mTORC1, as mentioned earlier. Therefore, a double safeguard system appears to exist to prevent high expression levels of AMAP1 protein, to prevent the cell-invasive phenotype from readily appearing, by targeting the 5’-UTR and 3’-UTR of *AMAP1* mRNA. Thus, normal-p53 appears to be an epithelial-specific safeguard preventing cell invasiveness, under conditions where mTORC1 becomes activated such as during active cell proliferation.

*MiR-96* and *miR-182* are first transcribed as a *miR-183-96-182* cistron, as mentioned earlier. The basic functions of miRNAs include coordination of the expression of various mRNAs, to orchestrate protein levels as required for specific cellular functions. In this regard, it is interesting to note that miR-96 also targets *Foxf2* and *Ezrin* mRNAs, to block the invasion and metastasis of lung cancers and renal cancers [29, 30]. miR-183 was also reported to be involved in inhibition of the invasion and metastasis of different cancers, such as lung, breast, and osteosarcoma [31–33]. On the other hand, normal-p53 may induce miR-200c to suppress the invasiveness of HMECs and MCF12A cells, in which miR-200c targets *ZEB1* to block EMT [2]. We found that miR-200c is not complementary to *AMAP1* mRNA and not notably induced by p53 in MDA-MB-231 cells (data not shown). Therefore, p53 appears to have various routes to suppress invasiveness, via different miRNAs in different epithelial cells. On the other hand, we do not know why cancer cells, such as MDA-MB-231 cells, have very low but detectable levels of *miR-96/182* expression even in the absence of intact p53 (i.e., leaky expression of *miR-96/182* in the parental cells and shp53 cells), whereas normal epithelial cells have undetectable levels of *miR-96/182* upon the loss of intact p53.

Our results indicated that the induction of miR-96 and miR-182 by p53 is an event specific to epithelial cells. Such epithelial cell-specific miRNA induction by p53 appeared to be predetermined by the epigenetic regulation of these miRNA gene loci. Moreover, not all miRNAs that are under the control of p53 in epithelial cells are specific to epithelial cells. Thus, our results give rise to the following questions: 1) when and what determine the epigenetic regulation of certain miRNAs, such as the *miR-183-96-182* cistron, to confer p53 the ability to block invasiveness in epithelial cells; and 2) whether or not epithelial cells increase *AMAP1* mRNA levels in the presence of intact-p53, to gain high invasiveness, such as during EMT, in addition to the mTORC1-mediated translational upregulation of this mRNA.

## Additional files

Additional file 1: **Figure S1.** Correlation diagrams between each miRNA and AMAP1. Correlation diagrams between expression levels of miRNAs shown in Fig. 2b and *AMAP1* mRNA. Correlation diagrams of *miR-96*, *miR-182*, and *miR-301a* are shown in Fig. 2c. (PDF 1029 kb)

Additional file 2: **Figure S2.** Epigenome status of each miRNA. The ENCODE data of miRNAs in Fig. 2a are shown by the UCSC Genome Browser. Definitions of the colors are given at the top of Fig. 3c. (ZIP 21369 kb)

## Abbreviations

EMT: Epithelial-mesenchymal transition
ENCODE: Encyclopedia of DNA elements
miRNA: Micro ribonucleic acid
mTORC1: Mechanistic target of rapamycin complex 1
RT-qPCR: Real time quantitative polymerase chain reaction
TCGA: The cancer genome atlas
TOP: Terminal oligopyrimidine
UTR: Untranslated region
