# Supplementary figures and images for: Epithelial-specific histone modification of the miR-96/182 locus targeting AMAP1 mRNA predisposes p53 to suppress cell invasion in epithelial cells
Source: Cell Commun Signal. 2018 Dec 4;16:94. doi: 10.1186/s12964-018-0302-6 (PMC6278066; doi:10.1186/s12964-018-0302-6)

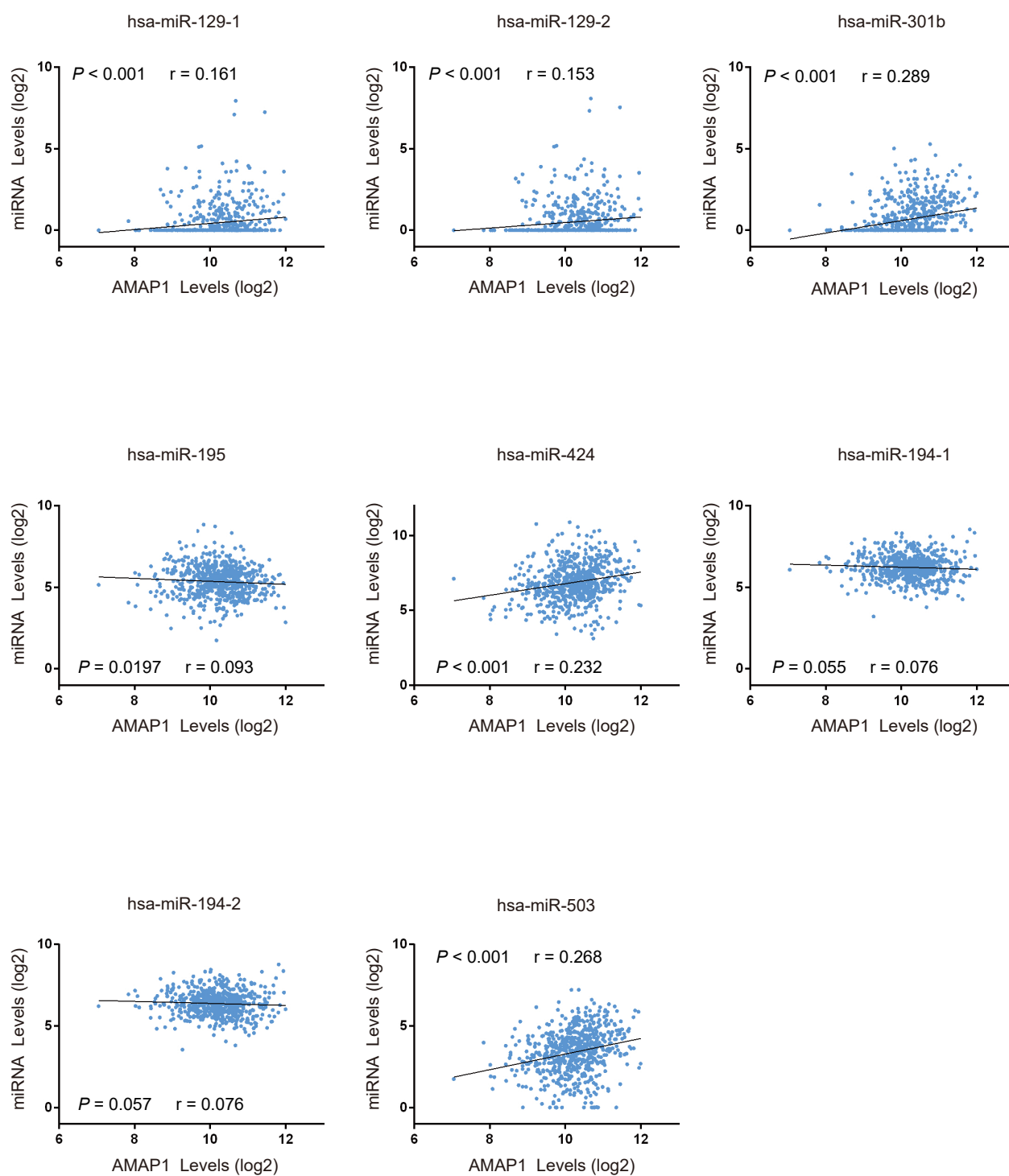

**Figure S1 Handa et al.**

Supplement: Supplementary file 1 — Figure S1. Correlation diagrams between each miRNA and AMAP1. Correlation diagrams between expression levels of miRNAs shown in Fig. 2b and AMAP1 mRNA. Correlation diagrams of miR-96, miR-182, and miR-301a are shown in Fig. 2c. (PDF 1029 kb) [file 12964_2018_302_MOESM1_ESM.pdf]

hsa-miR-4745

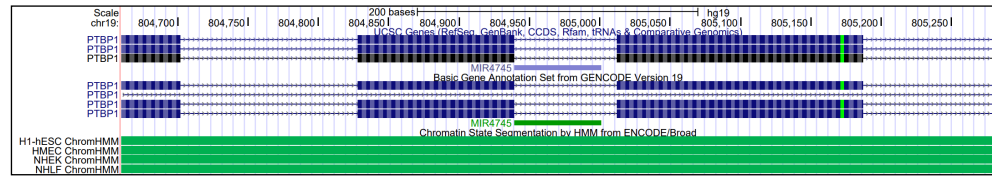

hsa-miR-503

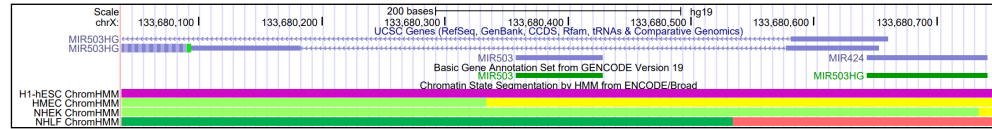

hsa-miR-642

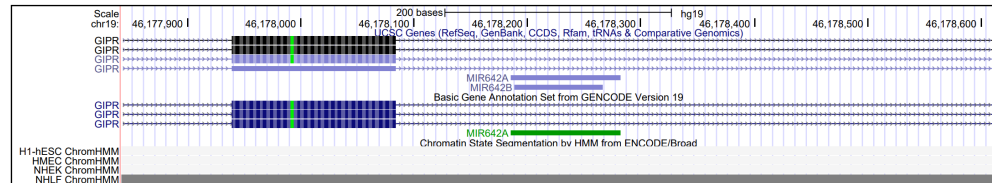

hsa-miR-663

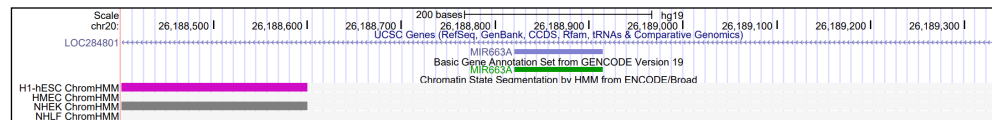

hsa-miR-769

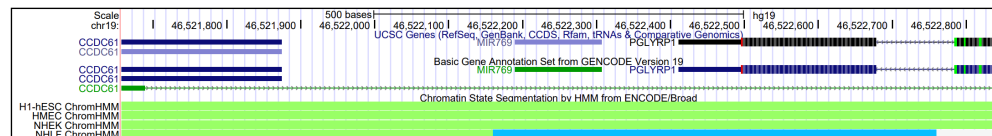

Figure S2 Handa et al.

Supplement: Supplementary file 2 — Figure S2. Epigenome status of each miRNA. The ENCODE data of miRNAs in Fig. 2a are shown by the UCSC Genome Browser. Definitions of the colors are given at the top of Fig. 3c. (ZIP 21369 kb) [file 12964_2018_302_MOESM2_ESM.zip › Handa-2 Sfig_2-3.pdf]
