# Supplementary material for: Epithelial-specific histone modification of the miR-96/182 locus targeting AMAP1 mRNA predisposes p53 to suppress cell invasion in epithelial cells
Source: Cell Commun Signal. 2018 Dec 4;16:94. doi: 10.1186/s12964-018-0302-6 (PMC6278066; doi:10.1186/s12964-018-0302-6)
Supplement: Supplementary file 2 — Figure S2. Epigenome status of each miRNA. The ENCODE data of miRNAs in Fig. 2a are shown by the UCSC Genome Browser. Definitions of the colors are given at the top of Fig. 3c. (ZIP 21369 kb) [file 12964_2018_302_MOESM2_ESM.zip › Handa-2 Sfig_2-1.pdf]

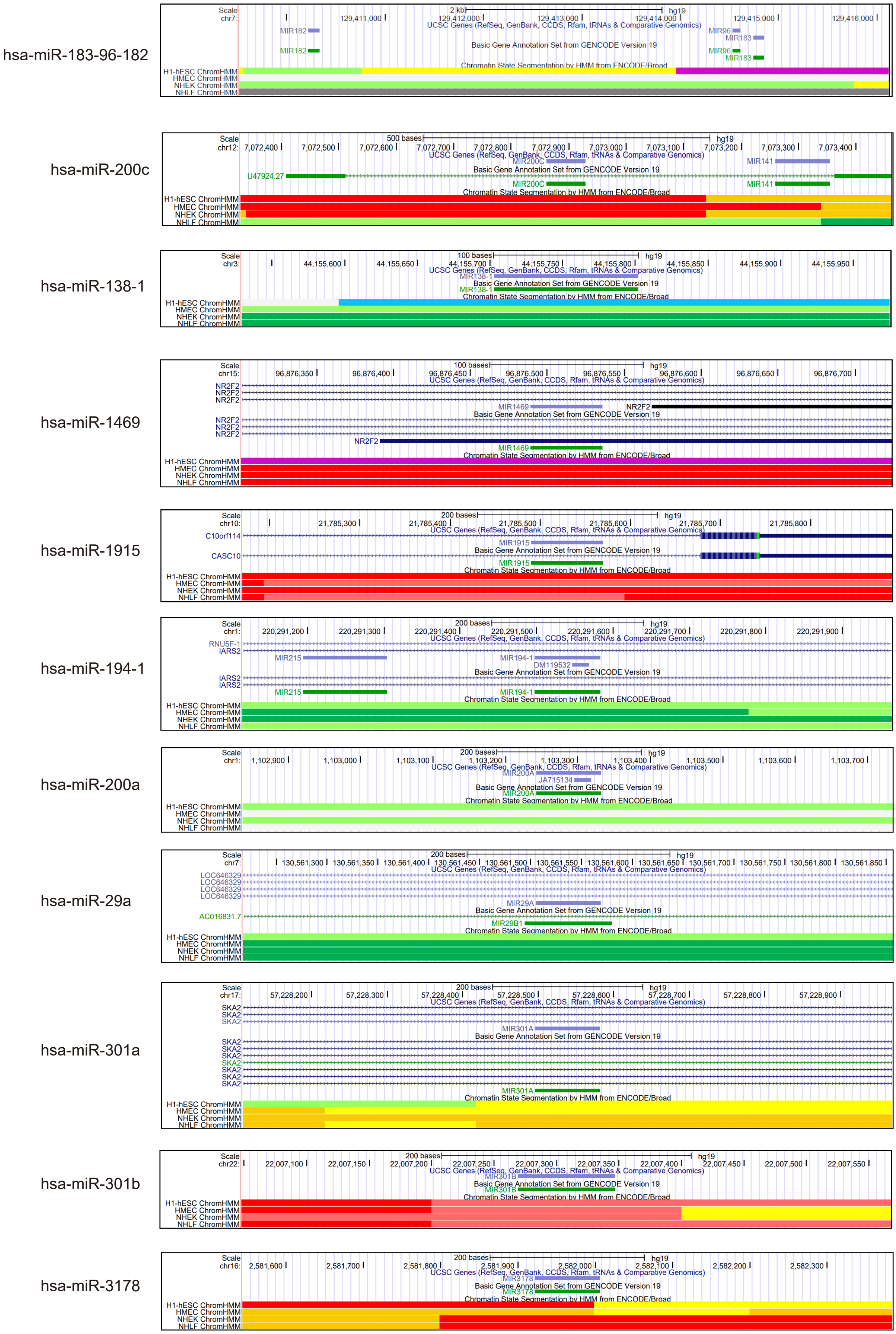

Figure S2 Handa et al.
